# Current Grief Support in Pediatric Palliative Care

**DOI:** 10.3390/children8040278

**Published:** 2021-04-04

**Authors:** Taryn Schuelke, Claire Crawford, Rachel Kentor, Heather Eppelheimer, Cristina Chipriano, Kirstin Springmeyer, Allison Shukraft, Malinda Hill

**Affiliations:** 1 Department of Pediatric Palliative Care, Texas Children’s Hospital, 6621 Fannin St., Houston, TX 77030, USA; cacrawf2@texaschildrens.org (C.C.); kmspring@texaschildrens.org (K.S.); 2 Department of Pediatrics, Baylor College of Medicine, Psychology Service, Texas Children’s Hospital, 6701 Fannin St., Houston, TX 77030, USA; rxkentor@texaschildrens.org; 3 Memorial Hermann Pediatric Hospice, 902 Frostwood Suite 288, Houston, TX 77024, USA; Heather.Eppelheimer@memorialherman.org; 4 Bo’s Place, 10050 Buffalo Speedway, Houston, TX 77054, USA; cristina@bosplace.org; 5 Department of Pediatrics, Pediatric Advanced Care Team, Atrium Health’s Levine Children’s Hospital, MEB 415-F, 1000 Blythe Blvd, Charlotte, NC 28203, USA; allison.shukraft@atriumhealth.org; 6 Justin Michael Ingerman Center for Palliative Care, Children’s Hospital of Philadelphia, 3401 Civic Center Blvd., Philadelphia, PA 19104, USA; hillme@chop.edu

**Keywords:** grief, bereavement, palliative, pediatric, death, support, programing, hospice, hospital, thanatology

## Abstract

Grief support changes as more is learned from current grief theory and research. The authors provide a comprehensive overview of current grief support as it relates to Pediatric Palliative Care (PPC). The following aspects of grief are addressed: (1) anticipatory grief: the nondeath losses that occur with a complex and chronic illness, as well as the time leading up to death; (2) grief around the time of death: the intense and sacred experience of companioning with a dying child; (3) grief after death: supporting bereavement and mourning through programing and other methods; (4) innovative approaches: the future of grief support. The contents of this article are meant to support and educate programs currently providing grief services and those aiming to begin the meaningful work of grief support.

## 1. Introduction

Growth in the field of Pediatric Palliative Care (PPC) combined with innovative grief theory creates new opportunities to support the bereaved. A death-avoidant culture breeds anxiety for families of children with life-threatening illness [1]. The mere thought of a child dying is overwhelming and unnerving. This experience can feel similar to waiting for a bomb to detonate: the dreaded anticipation of watching a timer tick down the seconds, the careful manipulation of wires to stave off the worst, the fearful waiting when all efforts return void, and the excruciating moments at the end. Then comes the aftermath of shockwaves rippling out, forever affecting each life it touches. Grief is heavy and isolating [2]. Families consistently report feeling abandoned by the medical system after their child dies [3,4]. Some describe feeling similar to aliens living on a planet continuing to revolve even though their world has shattered. There is no map for this uncharted territory, which is highly personal and often lonely. Indeed, it is the duty of providers to walk alongside the grieving, helping to clear a path and strengthen recovery as their lives move forward [5].

Children with complex and chronic illnesses can be referred to PPC teams for many reasons: complex medical decision making, pain and symptom management, longitudinal support, and/or end-of-life care. While not all children who utilize PPC services are nearing the end of their life, some do live shortened lives [6]. PPC teams are specially trained to care for children and their families facing these difficult circumstances. The teams typically include physicians, nurses, social workers, chaplains, grief specialists, psychologists, administrative support, and sometimes child life specialists [7]. One of the many benefits of utilizing PPC services is the grief and bereavement support that surrounds the death of a child. This multifaceted, interdisciplinary, and holistic grief support begins well before death and continues throughout the trajectory of grief, based on each family’s specific needs [8]. Academic literature and anecdotal family feedback are clear about the need for comprehensive bereavement efforts [3,7]. Despite understanding the importance of comprehensive bereavement care, providers may not know where to start, what is working, and what is most impactful for families [9]. The authors hope that the contents of this paper will provide a practical overview of grief and bereavement care to bolster world-wide initiatives of support. The school of thought is this: one person cannot do everything, but everyone can do something meaningful for each grieving person.

The following paper is divided into three categories of grief support: anticipatory grief, grief around the time of death, and grief after death. Each category addresses the important nuances of care when a child dies. The authors highlight what has been most impactful in providing support during these critical times to children and their families. All interventions are based on up-to-date research, theory, and best practices, keeping mind the importance of family systems, culture, and spirituality. Anecdotal responses from children and their families are included, some bearing actual names approved with permission. A call to action and innovation concludes this article, encouraging providers to continue the good work toward a more grief-healthy world.

## 2. Anticipatory Grief

### 2.1. Loss of Normalcy, Control, and Worldview

The diagnosis of a life-limiting condition begins a family’s grief journey. Suddenly the hope for a “normal” childhood is no longer possible as the child faces tests, treatments, procedures, and more. Caregivers begin asking *“What if…”* questions, and typically push the thought of death away. Everything feels new and foreign, and any semblance of control disappears into the hands of medical teams. Many families face the loss of support systems, jobs, financial security, physical wellbeing, faith, mental health, and more. These secondary losses compound the stress of a child becoming hospitalized [10]. Feelings of safety fade away as the prior assumptive world cracks open, meaning each person grapples with what is good, just, and meaningful [11]. Losses abound as the child moves through their diagnosis and care toward the end of their life. Throughout this experience, the family works to cope with their reality, striving for resilience and holding many hopes at once. It is in these moments that PPC can be most helpful.

Best practice encourages consultation of PPC teams earlier in the diagnosis trajectory instead of near the end of life, in part due to the concurrent care model [12,13]. In 2010, the passage of the Affordable Health Care Act included Section 2302, allowing children covered by public insurance to receive concurrent hospice services and disease-directed therapy [14]. Early interactions naturally provide more time to address any grief building up throughout the illness trajectory. Helping families develop a framework of coping early can provide a sturdy foundation for the more difficult moments ahead [15]. The PPC interdisciplinary team is educated on current grief theory and practice, which allows for care to be tailored toward addressing these issues. Sometimes families do not recognize that their difficult emotions, outbursts, and physical ramifications can be attributed to their underlying grief. Validating and naming this grief can allow for a new perspective and potentially a gentler personal outlook. When the time comes for complex medical decision making or advance care planning, this foundation of coping can promote more clear-minded thinking [16].

Anticipatory grief typically begins soon after the medical team shares: “We have exhausted all medical interventions. We are deeply saddened to say there is nothing left that will cure your child” or when families see their child’s physical or mental signs of decline. Families often worry that the medical team will stop providing care as the child nears end of life. However, this emotional time is when specialized PPC symptom management and compassionate grief interventions take priority [17]. For some families, the child is old enough and/or awake enough to experience the anticipatory grief of their own death. Additionally, families need support with incorporating siblings into end-of-life care. The support offered during this time is crucial to the trajectory of grief for the family. The following sections highlight each of these psychosocial interventions.

### 2.2. Death Anxiety

Though dying elicits heightened emotionality, assuming that all children will be highly anxious in the period immediately preceding death is both inaccurate and undermines effective assessment and treatment of symptoms [18]. Factors that may contribute to heightened death-related anxiety include restricted or veiled information about one’s health status, limited involvement in medical decision making, and fear of poorly controlled physical symptoms (e.g., whether it will hurt when the child dies). Adolescents are more likely than younger children to experience anxiety and depressive symptoms at end of life [19]. Death anxiety may also reactivate and/or exacerbate premorbid anxiety disorders, such as panic disorder or generalized anxiety disorder [18].

Poorly managed anxiety toward end of life is one of the most common and most troubling symptoms of suffering identified by recently bereaved parents [20]. Research demonstrates that unmanaged child anxiety at end of life is associated with poorer psychological outcomes for parents after death. In one study, parents whose children’s emotional distress and overall well-being were more impaired demonstrated more psychological problems up to 9 months following the death [21].

One aspect of death anxiety that may be particularly difficult to assess and treat is the cyclical relationship between organic physiological symptoms and anxiety-associated bodily sensations. Dyspnea, for example, is commonly experienced by individuals toward end of life. This sensation can physiologically mimic activation of the sympathetic nervous system (i.e., “fight or flight” response), which may trigger a cascade of anxious thoughts (e.g., “Am I dying right now?”) and further physiological arousal. Similarly, there are several sympathomimetic drugs (e.g., prednisone, dexamethasone, bronchodilators) prescribed to alleviate physical symptoms that may inadvertently worsen anxiety [18]. Some adolescents and young adults wish to be prepared with knowledge of how they will likely die (e.g., gradually becoming more tired until one day he or she will not wake up, versus experiencing an acute event). This knowledge may decrease anxiety in some domains while potentially elevating anxiety in others. For example, an 18-year-old young woman with end-stage lung disease secondary to cystic fibrosis was experiencing frequent anxiety about having an acute medical event, which led to her avoiding being in public or even momentarily separated from her mother. When she learned about the expected gradual course of her decline, she became more comfortable engaging in her life without persistent fear; however, she also began experiencing increased anxiety when going to sleep at night out of fear she would not wake up in the morning. Although her nighttime anxiety increased, this anxiety could be directly addressed and her specific fears managed (e.g., collaboratively generating a bedtime routine that incorporated relaxation strategies while also providing opportunities for physical closeness and open expressions of love with her family).

Some youth demonstrate “protective avoidance” and denial of the seriousness of their condition which can, in turn, impair coping ability and make it more challenging to adequately address their fears and anxiety [18]. In addition to fears about their own suffering, children and adolescents often worry about how their loved ones will cope following their death [22,23,24]. This can hinder their willingness to openly express their own distress. PPC teams often encourage families to tell the dying child that while they will be sad after the child’s death, they survive and carry on the child’s legacy. With the help of a PPC team, one family told their 12-year-old son nearing the end of his life “We will miss you more than we can say, but we promise we will be ok”. The child died within hours of the family giving this permission to die.

Notably, death anxiety must be considered within the context of family and cultural values. There are several cultural influences on communication practices (e.g., open communication about illness-related information), involvement of children in medical decision making, pain expression, and attitudes toward suffering, all factors which are seemingly known to differentially influence a child’s anxiety [18,25]. While some of these may be identified as contributing to a given child’s anxiety, intervening without appropriate cultural contextualization and alignment is inappropriate and may be harmful.

### 2.3. Legacy Building

When thinking about legacy building, many practitioners often focus on the tangible items that can be cherished by siblings and caregivers before and after their child’s death [26]. In PPC, through multidisciplinary team collaboration, many providers currently offer tangible legacy building items including hand impressions, ink prints, lock of hair, ink print jewelry, and heartbeat recordings. Many of these tangible keepsakes have been studied and reported to be beneficial to caregivers and siblings in their grieving process [27].

These keepsakes are often created when the child is actively dying or even after death, which focuses more on the time of death instead of the child’s life. When a child is nearing the end of their life or has died, the family experiences complex emotions and may not be able to focus on the choice of the keepsakes offered. They may decline services due to clouded emotions, wanting to leave the hospital or items associated with the death of their child instead of their child’s life and true legacy. This practice risks depriving the family of positive associations with their keepsakes. Instead of offering keepsakes closer to the time of death, the emphasis should remain on honoring the child and family through focusing on the context in which we provide legacy building services.

The PPC field must move forward and consider the importance of intangible legacy building. Beyond tangible keepsakes, there is a duty to focus on the wishes of the child and family and to consider what favorite activities or milestones are of importance to them [28]. In addition to those tangible keepsakes, there should be a focus on a living legacy perspective, which can give healthcare teams a renewed sense of honoring the life, living wishes, and legacy building opportunities for the children and families PPC teams serve [29]. This requires investigation of future hopes and wishes of the child, their sibling(s), and caregivers. Providers may ask questions such as: What do you want to have others remember about you and your life when you are gone? What is of importance to you—is it gifting personal items to others once you die, is it making a personalized drawing or creating videos for your loved ones?

For a dying child, autonomy is crucial as they may already be losing their sense of autonomy due to their life limiting diagnosis impacts on their physical health and capabilities. Therefore, intangible memory making and living legacy building can be poignant psychosocial interventions. A living legacy is focused on the life of the dying child and encourages active participation in the creation of keepsakes which allows the dying child to have control in a situation where they cannot change the outcome. Additionally, creating legacy projects can be beneficial for a dying child and aid in easing anxiety regarding the burden of leaving their loved ones behind when they die and instead reframe these emotions towards creating loving gifts that are personalized to important people in their lives. A dying teenager voiced the concern that their younger sibling would “forget me because he is so young” and this is an important reason to create personalized legacy building items specifically for siblings. Including siblings in legacy building projects and focusing on ways for the dying child and sibling to connect is crucial. Teams often create one memory box for an entire family but forget that perhaps a sibling would like a memory box of their own so they can remember the unique bond and relationship they had with their sibling.

### 2.4. Creative Modalities

#### 2.4.1. Creative Therapies

Creative outlets can benefit both the child and family. Expressive therapies such as music therapy, art therapy, and play therapy provide opportunities for processing intense emotions and experiences in grief [30]. These therapists have extensive training in their specialties and can enhance coping and meaning at the end of a child’s life. If a PPC team has access to these therapies, they should be consulted to provide their assessment and intervention.

Music therapists utilize sound and rhythm to improve distressing symptoms at the end of life (e.g., pain, anxiety, depression, shortness of breath, mood) [31]. Engaging PPC children and families in creation of music, playing instruments, has shown positive outcomes in the end of life (EOL) experience [32]. Additionally, the music created during these therapy sessions become legacy items for the family after the child’s death.

Art therapists partner with the dying child and their family to create art through various mediums with a therapeutic purpose [33]. Allowing choice of modality within the creation process provides control at a time when finding control is difficult. One child utilized art therapy to explore his death anxiety, likening it to a deep ocean, through painting and clay figures. This allowed him to process his fears and reach a calmer state of mind before he died.

Play therapy can be especially helpful as children process their complex experiences and emotions [34]. The play therapist can facilitate a therapeutic, safe environment in which the dying child and/or their siblings can experience symbolic play. These interventions can promote belonging and attachment within the family unit, leading to healthier coping in grief.

#### 2.4.2. Applying Creative Concepts

Not all PPC teams have access to these specialties, therefore these creative concepts can be incorporated on a primary level. For example, the PPC provider can discuss with the family what sounds or songs might be soothing to hear as the child nears the end of life. That music can then be played at the bedside to promote a healing and peaceful environment. Families can utilize art as a means for family bonding and coping, incorporating each person’s art contribution into a comprehensive piece which will endure for years to come. Play is the work of children [35] and allowing the dying child (if possible) and/or siblings to play out their experiences near the end of life can be a highly therapeutic form of expression [36].

Legacy work should always be based on cultural and religious considerations and be personalized to each child and family [37]. PPC providers can facilitate conversations and storytelling with families to engage in better understanding who the dying child was prior to their diagnosis to develop ideas for creative legacy work. They may ask to see photos or videos of their child engaging activities they loved such as dancing, singing, or playing a favorite sport. Understanding the dying child’s personality outside of the diagnosis facilitates more meaningful creative legacy building.

### 2.5. Preparing and Planning

For many families, the death of their child will be the first time they have planned a funeral or experienced loss of a closer family member. For this reason, it can be helpful for social workers, Certified Child Life Specialists, chaplains, or other specialized providers to guide them in memorial planning [38]. Many families ask about logistics at the hospital, wanting to know what will happen from the time their child dies until they are transported to the funeral home. These providers can provide an overview of basic choices families may be asked to make. For example, some parents choose to bathe their child’s body, some families stay for an average of 2–3 h with the body, telling stories and saying their goodbyes, and some parents are not comfortable being present through the end of their child’s life. For families who choose home hospice, finding a safe place for siblings to go in the moments they feel overwhelmed can be helpful. They validate families’ choices, remind them that the child will be treated with love and dignity, and reassure families that the child will not die alone.

Social workers and child life specialists may also help families with the choice of funeral home, as well as some basic planning information. It can be helpful for parents to appoint an extended family member act as the point of contact for the funeral home. Part of the contact’s job can be to reach out to a funeral consumer organization (such as Funeral Consumers Alliance in The United States [39]) to provide information about how to negotiate reasonable prices for funerals and what is required after death under state law (i.e., burial vaults, embalming, use of a funeral director, etc. all required by your state, region, or country). It can be helpful to inform the family that cremation is typically less expensive than burial, keeping in mind the family’s cultural and religious preferences. Many social workers can also find limited financial assistance for pediatric funerals through national and international disease-specific organizations, regional and local charities, and occasionally national and international charities specific to assisting families with the cost of pediatric funerals.

### 2.6. Considerations for International Families

When treatment options for complex illnesses grow increasingly limited, families often search outside their own states or countries to engage in more experimental therapies or phase I trials [40]. The decision to seek international treatment is often accompanied by feelings of fear and hope but also includes many unanticipated financial and emotional consequences, particularly if the treatment is unsuccessful.

To mitigate these complications and empower families to make informed decisions, it is important for the child’s treatment team to discuss prognosis and anticipated effectiveness honestly and regularly. The option to return home should always remain available to the extent that the child is able to travel with assistance from care managers and the institution’s international office, which can collaborate with the embassy. Providers can sometimes identify a “window of time” in which the child may safely travel; families should be advised when this window may be closing alongside the risks and benefits of remaining at the treating facility.

Despite innovative treatments, some children die outside of their home countries because they were temporarily or semi-permanently in another country to receive care and either could not or chose not to return home before the time of death. Families often face significant financial and logistical burdens when they choose to return their children’s remains to the home country, particularly if they choose burial. In addition to the cost of ground or air transportation of the body, many countries require a fee that many families cannot afford to allow the body back into the country [41]. Though families may prefer burial, those with limited income or financial support are often advised to cremate remains, which can be carried across many borders with no or minimal fees.

Families and providers must work with their embassy, a local funeral home, and a funeral home in their country to coordinate international transportation of remains. Some embassies will assist with transportation planning and costs for its citizens [42]. Families of children who die within the home country but across local borers may similarly need to choose funeral homes in the city where the child died and the city where the child will be buried to coordinate transportation. For families following the Muslim or Hindu faith traditions, these collaborative efforts will typically need to be expedited to facilitate burial in the home country within 24 h after death. Setting expectations of prognosis alongside honest ongoing discussions of how the family will eventually return home helps the family make realistic plans for location of death [43].

### 2.7. Spiritual Pain and Suffering

Children and their families often seek to participate in unique spiritual, cultural, or familial traditions as they near end-of-life. Examples might include engaging in a specific type of prayer, consuming herbal substances for healing, opening windows to allow the soul to exit the building peacefully, and others. Providers may ask questions such as, “What is most important to your family as your child nears the end of his life?” or “Is there any type of ritual or practice that would be meaningful to you at this time?” to facilitate such practices.

The experience of a child’s anticipated death challenges one’s comprehension of God’s presence and power, and it can be difficult to understand the meaning of a child’s spiritual pain and suffering. When children face mortality, spiritual care enables children to consider existential questions such as, “Why me? Where is God in all this? How can this happen?” and thoughts such as, “I’m too young to die”. Identifying spiritual and religious beliefs guides the chaplain’s ongoing anticipatory grief assessment, which informs the biopsychosocial plan of care. A biopsychosocial plan of care focuses on the interconnectedness between biology (genetic, biochemical, etc.), psychological (mood, personality, etc.) and social (cultural, religious, spiritual, etc.) factors. The purpose of this model is to better understand health and illness.

#### 2.7.1. Case Presentation

Greta was a 17-year-old teenager with a congenital heart defect who had a heart transplant at a young age and is currently in heart failure. During her hospitalization, she learns that she was not a candidate for a second heart transplant. Greta did not consider herself religious (formal, structured, beliefs, practices, rituals) [44] but instead found comfort in praying to God through journaling, a tool that can provide meaning-making (which brings significance, purpose, and direction to people’s lives) [44]. When the chaplain met Greta, she shared she is devastated and angry at her God. Greta lamented, “Why me, God? Where have you been? Why don’t you do something? I’ve been praying for a new heart, but you are giving up on me. How do I pray now?” Greta expressed overwhelming anger and feelings of hopelessness, anxiety, and despair. She cried, “I’m not ready to die”. The chaplain actively listened to Greta’s anticipatory grief and feelings of abandonment.

In subsequent chaplain visits over the next few months, Greta’s anticipatory grief included a review of her life. Greta’s senior high school goal was to show her artwork in a gallery event, but with the progression of her heart disease this goal was not obtainable. Abandoning her hope of going to college, getting married, and being a mom was devastating. Greta had trouble with activities of daily living and grieved her independence. Greta said, “My life is being cut short. There is nothing left to live for” and doubted the value of her life. Greta wondered why God was allowing her to die young. The chaplain offered Greta a spiritual tool, poetry, as an activity to identify what brought Greta strength, peace, and security.

At another visit, Greta’s anticipatory grief included sharing her bucket list. She wanted to travel to art museums to explore ideas for her legacy. Greta stated that while she still believed in God, she had difficulty praying. The spiritual exercise revealed that Greta’s strength came from her family and God because they were the only constants in her life. Greta’s peace was in her journaling, and her security was in knowing she would be with God when she died.

During Greta’s last hospital admission, Greta realized she was not getting better. Greta forgave and thanked God for allowing her to live longer than anyone anticipated. Greta wondered what it would feel like when she was dying—“Will it hurt? Will I fall asleep?”. Greta determined that she was not afraid to die because God would welcome her into eternity. Sharing a dream, Greta described eternity, where she could breathe and had the energy to paint. She met her God, who assured her everything would be alright. Greta wondered if her family and friends would remember her, and she decided that her legacy was to give her artwork away to close friends and family. Greta hoped her family knew how grateful she was for all the care and love they shared and was at peace with the decision to have home hospice for the end of her life. Greta died peacefully at home, surrounded by her twin brother, parents, and other family members.

Understanding age-appropriate anticipatory grief reactions and conceptions of religion and spirituality is essential when assessing a child’s response to terminal illness. Adolescents such as Greta have an adult-level understanding of death, enabling them to face and deal with their life’s challenges. They can think abstractly and are often curious about death’s existential implications [45].

Healthcare professionals working with children with life-limiting illnesses must evaluate spiritual pain and spiritual suffering. Using different strategies that make sense for each child (religious and spiritual practices, meditation, art, physical exercise, group therapy, etc.) contextualizes pediatric patients and their families.

#### 2.7.2. Spiritual Pain

Spiritual pain is the pain that comes from the “hidden” areas of one’s life. While not found on a pain scale, spiritual pain is still authentic and can impact a patient’s physical and emotional health.

There are four areas of spiritual pain:

1. Meaning is the struggle to make meaning of one’s life (relationships, the world around them, e.g.). In Greta’s case, meaning included communication with her friends, lamenting God, and not fulfilling her life’s goals of having an art show, being an art teacher, getting married, and being a mom.
2. Forgiveness means the pain of forgiving others, themselves, and their God. Greta forgave God for feeling abandoned and was thankful to exceed her life expectancy.
3. Relationships with others refers to whether interpersonal relationships are positive or negative. Greta hoped her family and friends would remember her fondly and be aware of her gratitude for them.
4. Hope represents the feeling that there is no hope or it doesn’t exist [46]. Greta realized she will not get a heart transplant and let go of her hope for a long life.

#### 2.7.3. Spiritual Suffering

Spiritual suffering arises when needs are unfulfilled. Just as physical pain signals an injury to the physical body, spiritual suffering indicates that one or more spiritual needs are threatened or unmet. While physical suffering can include a threat to one’s mobility, spiritual suffering has threats to one’s beliefs and purposes in life [47]. The experience is known as spiritual dissonance, where the dissonance lies between one’s faith and critical events happening in one’s life. Spiritual suffering is found clearly in those difficult times when individuals cannot find sources of meaning, hope, love, peace, comfort, strength, and connection in life [48].

Spiritual suffering surfaces when children prepare for their death. The following statements and thoughts demonstrate various aspects of Greta’s spiritual suffering:

- Concern about life after death: “Will it hurt to die?”
- Loneliness: “None of my friends have time for me anymore.”
- Fear of not being remembered: “I’m worried that everyone will forget me.”
- Anger towards God or others: “Why did God let me have a heart that doesn’t work right? It’s not fair.”
- Desire for a relationship with God: “Where is God when I need God most?”
- Finding no value in life: “I haven’t done anything that makes me proud.”
- Loss of future, relationships, self, and health: “I’ll never be an art teacher or get married.”
- Feeling of guilty for doing something wrong: “I didn’t pray enough; that’s why I didn’t get a heart.”
- Need to resolve unfinished business: “I wish my family knew how grateful I am for their care and love.”
- Separation from peers or their community: “I’m too weak to go to school and paint.”
- Questioning belief systems/rituals: “My friends and family pray for me, but I’m not getting better.”
- Struggling to find meaning: “I don’t understand why this is happening to me” [49].

Spiritual interventions such as poetry aim to ease, resolve, or reconcile spiritual pain and suffering so children may experience peace, reconciliation, wholeness, and comfort [50].

#### 2.7.4. The Nuance of Spirituality

For children with life-threatening illnesses and their families, spirituality ebbs and flows. From diagnosis to death, the journey may be hopeful one day and disappointing the next. Sometimes the setbacks are sudden and significant; other times, awareness gradually changes by steps. As a child’s physical status changes, the aspects of their spirituality often (but not always) simultaneously shift [45]. Religion and spirituality form the basis of meaning and purpose for children and families. In the biopsychosocial model of care, spiritual pain and spiritual suffering need to be addressed. It is difficult to know what to say when children ask deep questions of life because there are no real answers. The Gretas of this world long for the chaplain and all providers to sit with them and support them in their struggles.

### 2.8. Deciding Where to Die

Where and how a child dies can hold great meaning for families, even years following the death [50]. While not all families get to choose where their child will die, providers can ask families about their preferences if there is time to plan. Children typically die either at home, at an inpatient hospice unit, or at a hospital [51]. Some families value a home death because it allows the child to be in a familiar, comfortable setting with the people they love. Home hospice can often offer support to ensure that the child’s symptoms (physical, emotional, and spiritual) are addressed. Some families do not want a home death, often because they do not want siblings to witness the death or fear that the home will be too difficult to live in after the child has died.

Some children are unable to go home to die because they are too ill (particularly when they need mechanical ventilation), but their families would prefer death in a setting more similar to home. These families may choose for the child to die in an inpatient hospice unit, which typically has comfortable beds, space for families to gather, access to the outdoors, and no monitors to distract from being with the dying child. Some hospice organizations can arrange for life-sustaining therapies to be discontinued once the child arrives at home or the inpatient hospice unit if the child is not stable enough to transfer without mechanical support [52].

Finally, many families choose an inpatient hospital setting as the place of death. This setting provides intensive care that can provide curative therapy, life-saving treatments, and comfort-based care in one place. Some families value the idea of “fighting” until the end of life, which may include resuscitation at the hospital [53]. Others focus on providing medicines to keep the child comfortable and/or remove life-saving treatments that are no longer helpful. Since children often receive treatment at hospitals for many years, they may feel comforted as they are surrounded by familiar providers near the end of life. The choice of where to die is deeply personal and can provide some amount of control to families during an extremely difficult time.

### 2.9. Family-Based Interventions before Death to Support Adaptive Grief

There is limited research evaluating the effectiveness of psychological interventions prior to death in mitigating complicated grief thereafter. There are known risk and protective factors, however, that can be addressed proactively. Family cohesion and the immediate caregiving environment are known to influence the grief process [54]. Preventative intervention, accordingly, may target facilitating emotional expression between family members, resolving core conflictual relationships, and helping families achieve or strengthen their sense of purpose and meaning from the anticipated loss [18]. Psychological intervention with youth receiving PPC services should include both children and their family members, with intervention becoming increasingly family oriented as the child declines.

There are a number of ways parents and providers can support siblings throughout the illness trajectory in order to promote more adaptive coping after the death of their brother or sister. Specifically, bereaved siblings desire early and ongoing psychosocial support throughout their brother or sister’s illness course, as well as more open communication about their disease and prognosis [55]. For children whose death is anticipated, siblings should be made aware of the impending death and prepared for what to expect (e.g., what their brother or sister will look like, what physical symptoms or events they may see). Siblings who do not have access to this information ahead of time demonstrate significantly higher anxiety, greater overall emotional distress, and lower social support long-term, as compared to siblings who are fully informed and involved [56,57]. Similarly to dying children themselves, siblings often refrain from talking about their own grief in order to shield their parents from additional distress, which in turn increases the likelihood of anxiety as time progresses [55].

### 2.10. Engaging Children in Death Conversations

#### 2.10.1. The Dying Child

Talking about death and dying with young children has been reported as one of the more challenging conversations for parents even when compared with conversations regarding other topics such as reproduction, ageing, and diagnosis of a serious illness [58]. Studies have shown that caregivers who are more anxious about an impending death were also more likely to avoid these crucial conversations and had a higher likelihood to utilize euphemisms when discussing death. These death euphemisms—including “they are passing away,” “they went to a better place,” or “sleeping”—often cause confusion for the sibling and have the potential to create new fears and stressors for a surviving child [59]. For example, a medical professional told one teenager that dying would feel like falling asleep. The teenager experienced so much anxiety around sleeping and fatigue that she required extensive psychosocial support and the addition of sleeping and anti-anxiety medication because she was afraid if she slept, she would never wake up.

Providers focus on concrete terms and encourage families to use the words “death,” “dying,” and “dead” with children. For younger children, children on the autism spectrum, and children with developmental delays it can be helpful to discuss the life cycle and the permanence of death. Children also benefit from discussion about the varied emotions surrounding grief and need to be reassured that it is appropriate to feel varied emotions like sadness, anger, relief, and happiness, and should be allowed to cry. PPC teams may prepare families for such conversations by asking questions like, “What if your child asks questions about death and dying? How would you like me to address these?” and offer to be a conduit to facilitate these conversations if they arise.

#### 2.10.2. Siblings

When speaking with caregivers prior to these crucial conversations regarding a child’s impending death, it is beneficial to ask questions about the sibling’s previous experiences with the death, including that of a family member, a pet, or friend. When exploring how caregivers have explained these events previously, one can ascertain wording that has been utilized previously and gain a baseline assessment of a caregiver’s comfort level with death and dying. The family might have certain terms they would like to avoid or cultural or religious beliefs they may want to incorporate into these conversations. Providers can additionally provide resources highlighting grief experiences at different developmental stages and what common terminology to avoid. Providers can remind families that all children, including infants and toddlers, experience grief and will perceive the emotions of those around them [60].

Another approach to encourage family’s participation in conversations surrounding death and dying is to discuss the positive long-term impacts these conversations can have on the sibling and caregiver’s relationship. Open and honest, yet difficult, conversations with their children help build a strong foundation of trust with their caregivers. Children often listen even when they appear focused on tasks; the pieces of conversations they overhear may impact their grief experience and even cause misunderstanding regarding the actual cause of a child’s death [61]. It is not uncommon for children to have beliefs that something they did or said somehow caused the dying child’s illness or even death. One bereaved sibling stated during a bereavement session “I thought because we were fighting to catch the ball and then he fell and bumped his head and ended up in the hospital (and was subsequently diagnosed with brain cancer) that I caused his brain cancer”. A teenager shared this concern in bereavement a session more than six months after her brother’s death. For this reason, it is imperative that PPC providers and family members reassure children that nothing they did or said caused the death of the child.

### 2.11. Assessing for Bereavement Risk

There are many validated tools available for assessing bereavement risk [62]. These tools can be helpful for providers hoping to tailor their resources and support interventions based on each family’s strengths and concerns. One such tool is the Modified Bereavement Risk Index (MBRI) [63]. Each family can be stratified as high-, medium-, or low-risk in bereavement. To use the MBRI, the provider (often a grief and bereavement specialist or social worker) gathers information about four areas of coping: guilt, anger, family support, and general coping needs. The intention of risk stratifying is not to label someone but rather to help initially guide the provider in how to best support the family. A parent who is high-risk might need more intense or frequent support, whereas a low-risk parent may not need support for as long in the grief trajectory. Each time a provider reaches out is another opportunity to assess risk for maladaptive coping. Just as in grief, the stratification may fluctuate throughout time, causing more intensive support months after from the death. Each PPC team can choose the validated tool that works best for their population.

## 3. Grief around the Time of Death

### 3.1. The Death Vigil

The days and hours before and after a child’s death are considered by many to be sacred. These moments represent the last time the family will be with the child and are likely to remain in the family’s memory permanently. This time, often referred to as a “death vigil,” may include any religious and cultural practices important to the child and family [64]. Many families choose to engage in quiet prayer, lay with the child, play music, or speak softly to the child to provide final words or sentiments. This is often a time when families give the child permission to die, letting the child know that the family will be sad but endure their absence. Providers should allow the family privacy during this death vigil unless the family requests the presence of specific providers. Providers should be near enough to the bedspace that the family can access them easily while maintaining enough distance that the family receives the privacy they deserve. It is beneficial for families to be notified about signs and symptoms their child may experience at end of life (e.g., agonal breathing, mottling, decreased urination, slowing of the gastrointestinal tract, decreased consciousness) [65]. Once these signs emerge, it is imperative for providers to increase their presence for facilitating additional symptom management and continuing education and psychosocial support for families. Often families are fearful of providing comfort medication as they fear it will hasten death [66]. Guidance from PPC or hospice providers can reassure families regarding the use of medication for appropriate pain and symptom management.

### 3.2. Postmortem Care

The moments after the death occurs are precious and sacred. For some families, this time is important for religious reasons. All preparations for special care and handling of the child’s body should take place prior to death to ensure these needs are met [67]. All families respond to postmortem care differently and families should be assured that each response is normal. Anticipatory guidance from providers can outline expectations for how the child’s body will begin changing after death so the family can decide how they would like to engage. Inviting the family to help bathe/wash, dress, hold, and spend time with their child’s body fosters moments of belonging and inclusion [68]. If keepsakes have not been made prior to the death (which is best practice, see Section 2.3), then providers can facilitate their creation with siblings and family. These sacred moments can be filled with many emotional responses, including agonal weeping, tearful smiles and/or laughter, peaceful storytelling, and angry avoidance; these emotional responses can be widely varied and should be normalized ahead of time as a normal part of the grieving process. The provider’s role is to validate and support the grief response and promote an environment of safety and welcoming care [69].

It can be very helpful for providers to have a protocol to follow during these times to provide every family a high level of care and resources to take home. Some teams utilize checklists in paper form or through the electronic medical record, which help ensure that all families are treated equitably and that staff can remember the many aspects of care at time of death. While specific methods to create comprehensive protocols may vary, development should be a collaborative and interdisciplinary effort to ensure success and consistency. While equity and consistency are essential to excellent patient and family-centered care, it is also important to provide a personalized touch, acknowledging the child as a person and bringing in unique aspects of their family and culture in any way possible [70]. The benefits of this level of care are immense for the family’s grief process. Families will remember in great detail what the medical team does and says during this time [71]. Providers should avoid clichés or any phrases that start with the words “at least”. Gentle handling of the child’s body always is recommended. Staff should consider how they would want their loved ones to be cared for to promote more empathy and compassion in their care.

Some institutions allow families to accompany hospital staff walking their child to the morgue. If the family chooses to go to the morgue, the child may be wrapped in a blanket to be carried. If the child is too large to carry, then the child can be covered while transported via stretcher. Prior to transportation, providers should determine a private and calming path to the morgue. Allowing the family to say goodbye at a time of their choosing can promote a smoother transition away from their child’s body [72]. If the family is having a difficult time with this transition, then offering constructive and gentle guidance can be helpful. Hospital staff can try saying, “In five minutes I am going to come and help you wrap him up so that I can take him to a safe place where his body will stay until the funeral home comes”. A similar phrase can be used for children who die with the support of hospice but tailored to the home setting. Sometimes the funeral home can pick up the child directly from the hospital room, which might provide some comfort to the grieving family. The goal is to orchestrate a peaceful separation from the child’s body.

Anticipatory guidance can also be provided for what will occur after their child’s body is taken away. Due to the complexity and intensity of grief emotions at this time, multiple methods of communication are important: verbal conversation, printed documents, and electronic or web-based information can reiterate important information. Siblings should also be included in these considerations and offered a safe space to seek refuge if they choose to not be present during removal of their sibling’s body. If possible, discussion of a potential autopsy, the process of transporting the child’s body to a funeral home, and the timing of it should all occur in person and prior to the child’s death. If the family’s grief response is too strong or timing is a barrier, the provider should reach out via phone call to the family the next day in addition to providing printed materials at time of death. The family should always have a clear person to contact for when questions arise. These details are crucial to the family’s grief response for the days, weeks, and months to come [73].

### 3.3. The First Weeks

In the first weeks after a child dies, families might find themselves in a state of shock and unable to process what has happened. Though grief is very personal and individual, common reactions related to the grief process may include the following, often fluctuating between them: (1) numbness and protest; (2) searching and yearning; (3) disorganization and despair; (4) reorganization [74]. Other emotions may arise, including disbelief and denial, guilt and self-blame, anger and helplessness, envy and resentment, and loneliness and yearning. They might additionally manifest physical symptoms of their grief, including: (1) shortness of breath, (2) weakness and fatigue, (3) restlessness and anxiety, (4) forgetfulness, (5) rapid heartbeat, (6) weight and appetite changes, and (6) tightness in the chest or throat [75]. Physical symptoms are likely to diminish over time, while emotional reactions will likely evolve as the griever begins to process their feelings.

## 4. Grief after Death

### 4.1. The Basics of Follow-Up Programing

According to an Evidence Based Practice Summary from Texas Children’s Hospital, there is a standard of bereavement care medical systems can provide to their grieving families [76]. The recommendation is as follows:

When a child dies, the family is left with an unwelcome, new reality. Many families experience a shift in their perspective in which they narrate their lives as occurring before and after their child’s death [3]. Families also experience compounding secondary losses; among them is the loss of support from the medical team, with whom the family has built trusting relationships [3,77]. Families may feel abandoned if the loss of contact is abrupt [77]. Some families also struggle to find the support they need after their child dies [3,77]. Bereavement follow up from the medical team can act as a bridge from the medical setting to reintegration into the family’s community [78]. While bereavement care is standard in pediatric hospice guidelines, it is not yet standard for pediatric hospital settings [78]. The literature demonstrates that the practice of bereavement follow-up benefits families, but the frequency and content of this support remains unclear.

The balance between a personalized and standardized approach should be assessed by each organization based on population and staffing abilities. At a minimum, the family needs a clear point-of-contact for any questions or concerns after death. Programing should span at least 13 months after the death to provide support during each of the first milestones e.g., the child’s birthday, holidays, the anniversary of the death [76]. Longer-term programming is also important as many families experience the need for support years after their child died. Sending personalized cards is a meaningful practice. These cards should contain authentic notes about how the child impacted each provider personally. A mail program that sends out supportive information at monthly intervals can also be helpful. Mail should arrive in envelopes that do not appear like medical bills, so that the family is more likely to open it. Additionally, calling the family a few days and months after the death to check in can be very supportive. On the whole, families appreciate hearing from their PPC and medical teams, knowing that people have not forgotten about them or their child [79]. Many organizations provide remembrance ceremonies, which is discussed in Section 4.5. Support groups can also be created and offered. Longitudinal support may include welcoming families on an advisory team or mentorship program years after the death of their child. Some families will reach out to create legacy programs in honor of their children (see Section 2.2). The programing should be multi-dimensional and include verbal, written, and electronic methods of contact to meet the family’s unique needs [80]. Not all families will find bereavement programing supportive, so offering discontinuation of any contact or changes in frequency of communication is respectful.

Most importantly, each family should be offered some form bereavement follow-up. Families need not feel abandoned by the medical system in their grief. Many organizations are willing to share resources and programing structures to help support PPC families nationally and globally.

### 4.2. Autopsy and Lingering Medical Questions

Often families have lingering medical concerns and questions about autopsy results after the death of their child. Sometimes final autopsy results take months to complete, which can cause distress for families seeking answers. Having clear communication about the autopsy process and how to receive medical records is imperative. The new onset of OpenNotes [81] for healthcare in the United States will allow families to see more intricate information in their child’s medical record than ever before. This will most likely lead to more questions that only the child’s medical team can help answer. Having a clear process in place to address these questions and concerns is crucial to the family’s grieving process [82].

### 4.3. Culturally Attentive Resources

When looking at the concept of bereavement, it is noted that the experience itself is a unique and yet universal one [83]. Yet, it is imperative that practitioners enter any discussion with a family with an awareness of their own experience with loss/grief and to have curiosity, not assuming based on the person’s culture or background that the experiences are similar. Instead, focusing on the person’s worldview helps orient the provider to that individual’s grief experience.

The concept of “worldview” as defined by Alesia Alexander Layne relates to the way in which people view the world, which effects coping and resilience in grief. There are three areas which all overlap and influence each other to compose this worldview. The areas are individual, universality, and culture. Individual is the elements that make people who they are, this could be their childhood, family, friends, beliefs or experiences. Universality is the experiences that all people go through, such as, milestones as we age. Culture is the internal navigation system that people have; it defines how that person will navigate the world that they live in [84]. An individual’s worldview is used to dictate how they will approach the bereavement experience. It is unique. Providers may find it easier to connect with people sharing similar worldviews, however diversity encourages everyone to expand understanding and empathy toward individual grief experiences.

As the population of the United States is shifting toward becoming more diverse, practitioners working with children and families who are bereaved need to practice cultural conscientiousness. This is composed of meeting the diverse needs of families from various family structures, abilities, sexuality, class, nationality, ethnicity, or spirituality by allowing the child or family to be the expert of their story [85]. This is a practice that reflects the principles of cultural humility, which incorporates the lifelong commitment by the practitioner to be learners and allow the child or family to be the expert of their own culture. It requires the practitioner to acknowledge that they do not know everything about another person’s culture and then showing respect by opening their hearts and mind to learning [86]. Given that bereavement is both unique and universal at the same time, practitioners must meet the child or family where they are and allow them to be the expert of their story.

### 4.4. Grief Support Groups

To set the context of bereavement groups and how they support a bereaved family’s grief journey, it is important to note that according to the Childhood Estimation Model of Bereavement in the United States, 1 in 14 children will experience the death of a parent or a sibling before the age of 18 [87]. In addition, the accessible that currently exist for bereavement groups or bereavement support it is necessary to acknowledge the availability of services. According to the National Alliance of Grieving Children, there are 733 providers for bereaved children that consist of bereavement centers, bereavement camps, hospices and individual providers in the United States [88]. Access to these providers is dependent on accessibility to major metropolitan cities as that is where most of these providers are located.

Bereavement groups for children often are designed around age, type of death and/or the connection to the person who died. In some centers, parents/adult caregivers group run at the same time as the children’s support group. In these groups, the general rule of “there is no right or wrong way to grieve” applies. According to Dr. William Worden, “grief is unique and universal” [83] which provides the opportunity for complete strangers to come together in a room and feel connected in their experience. Dr. Worden’s Four Task of Mourning [83] often provide a helpful framework for working with the bereaved and a basis of much of the programing and activities conducted at bereavements centers. Participants of bereavement groups can receive and give support to others who “get what it is.”

In the bereavement groups, activities are provided for children to be able to use various forms of expression (art, writing, music, play and/or physical activity) to share their thoughts, feelings and memories surrounding their grief journey while also learning health coping strategies to manage their reactions to the complex emotions of bereavement. At Bo’s Place, a bereavement center in Houston, TX, these various forms of expression are utilized in combination with a variety psycho-dynamic theories to create the activities that are used in the programing. [89] This would be during the first few meetings, providing an activity for children to begin to affective and cognitive process which could include drums and the instruction surrounding the activity would include when an emotion is mentioned, children are to play the drums to that emotion. Towards the end, the instruction could be given to play the emotion they felt when their person died [88].

Meanwhile in the adult groups, parents are often grouped with others who have experienced a similar death and the groups focus more on sharing ones experience while receiving and providing supports to fellow groups members. The length of stay within these bereavement groups varies on programing and organization. Some programs provide a time-limited option where a family attends collective for a set amount of time while other programs offer an open-ended option. Within the open-ended option, families are able to choose when they are ready to say goodbye. This allows the ability for individual families to choose if they will participate a few weeks, a few months or a few years [89].

A unique factor of many bereavement groups are that they are facilitated by lay-trained volunteers. Many of which attend an intensive training (ranging between 24–48 h), complete required observations and are placed with a cofacilitator who is experienced. The role of these facilitators is to truly be present and to facilitate the activities or the conversations. In some centers, facilitators are former participants and in others they are people who want to give back. For many families, a bereavement center is the only choice they have in seeking support as many of them operate free-of-charge [88].

In determining whether it is effective to utilizing bereavement support groups in the grief journey, it is helpful to look at participants’ satisfaction. At the end of their time at within the program at Bo’s Place 100% of participants would recommend Bo’s Place to another grieving family. Qualitative data has also supported statements that participants valued having the support of others in group [89].

It is often stated by children participating in these bereavement groups, “At school I am the only kid in my class, grade or school who has had a death. Then I come here and there are other kids who get it”, While the support groups are facilitated by lay-trained volunteers the support is provided mutually between the participants.

### 4.5. Memorial Events—Services and Ceremonies

Memorial events are meaningful opportunities for families to dedicate time to reflect, honor, remember and celebrate their children’s lives and legacies. Many organizations provide these services and ceremonies based on the child’s diagnosis or date of the child’s death. Successful memorial events incorporate a variety of components to make the events meaningful for families and staff, including music, inter-faith prayers, non-denominational, secular readings, and a slide presentation including the children’s photographs and/or names [90]. It is important to incorporate as many personal elements into the memorial event as possible, such as a photo slideshow, having the family say a few words, writing notes to their children, displaying each child’s name, etc. Depending on the number of families invited, the memorial events will need to accommodate this personalization in different ways. These types of offerings should be free-of-charge to the family if possible.

Family feedback after memorial events is overwhelmingly positive. Table 1 shows comments from a system-wide, multi-year remembrance ceremony. Even years after the death of a child, families seek out these special events to memorialize their child. One parent said, “I never stop thinking about her, but spending this uninterrupted hour loving (child’s name) helps me feel like her mother even now”. This is the heart behind remembrance programing: providing opportunities for families to grieve and love openly. These ceremonies also offer an invitation into community. Families are surrounded by others grieving a similar loss which can feel validating and provide solidarity. At these ceremonies, grief support pamphlets and support group flyers can be provided for families to take home, furthering outreach beyond the hour spent together.

Bereaved parents who return to the hospital for a memorial event value the opportunity to honor their child in a supportive setting. Parents report finding comfort in connecting with others. Children’s hospitals are encouraged to establish or further refine memorial events in support of grieving parents. According to Macdonald and colleagues [91], three themes emerged regarding parental experiences of staff members’ acts, i.e., (1) parents placed great importance on the hospital’s memorial service and on staff members’ presence at the service; (2) parents found it difficult to return to the hospital after the child’s death but all attended the memorial service, finding some closure in the return; and (3) parents appreciated receiving cards and greatly valued staff members’ efforts to telephone/visit and to attend the funeral. Months later, parents remembered positively which staff members engaged in which activities. Conversely, parents expressed disappointment when staff members did not engage in these activities and/or were absent from memorial/funeral services.

Efforts to support families and to commemorate deceased children are appreciated by bereaved parents. Staff members’ absences at commemorative events and a lack of supportive acts are noticed and regretted by families. Staff members and program administrators should attempt to arrange workloads to ensure meaningful contact between staff members and parents during the bereavement period.

### 4.6. Counseling and Therapy

Grief is a normative process in response to a personal loss. While incredibly painful, individuals’ grief experiences are generally not pathological; therefore, formal psychological intervention is not always indicated. Intervention is typically not considered until 6 to 12 months after the death of a loved one, so as not to interfere with or pathologize the natural grieving process. Grieving the loss of a child is particularly distressing, with bereaved parents reporting more intense and prolonged symptoms of complicated grief as compared to individuals grieving a spouse or parent [92,93]. Ten to twenty percent of parents will experience complicated and/or prolonged grief (i.e., symptoms and functional impairment that persist more than one year after death), particularly after the loss of a younger child [55]. The goal of intervention is not to “overcome” or forget about their loss, but rather to help the bereaved be able to remember their loved one with peace and find ways to maintain a continuing bond after death.

#### 4.6.1. Interventions for Bereaved Parents

Research suggests a pronounced underutilization of mental health services among bereaved parents [94,95,96]. Forty percent of parents in one study voiced desire for support but noted they did not pursue treatment [97]. There are a number of barriers to seeking care that are specific to grief, including reticence to accept reality, desire to avoid painful reminders of the loss, and a lack of awareness of the role and availability of mental health support in bereavement [55].

There are limited published trials evaluating psychological interventions for bereaved parents with complicated or prolonged grief. Research increasingly supports cognitive behavioral therapy (CBT) as an effective treatment [98]. Cognitive restructuring and exposure appear to be the core processes affecting treatment outcomes. Narrative therapy also demonstrates promise in addressing complicated or prolonged grief. Such intervention targets constructing meaning from the death and assimilating the death and loss memory into one’s personal narrative through journal writing and retelling the story of the death [99,100,101]. Across interventions, larger treatment effects are demonstrated in individuals with more severe grief symptoms at time of intervention [8].

#### 4.6.2. Interventions for Bereaved Siblings

Bereaved siblings report desire for increased bereavement support for a number of years following their loss, with specific support needs changing over time according to their cognitive and socioemotional development [56]. Psychosocial support for bereaved children can be generally classified into two categories. Grief camps are considered preventative interventions designed for the spectrum of bereaved youth, whereas psychological treatments target a subset of children with severe and persistent grief reactions [102]. Although there are limited studies evaluating the effectiveness of grief camps for siblings, preliminary research suggests such camps lead to decreased symptoms of anxiety and posttraumatic stress, increased coping skills, and decreased feelings of isolation [103]. This is achieved through allowing space for shared emotional expression and facilitation of grief work in a shared environment, often interspersed with play and recreation that allows for distraction and creative, developmentally appropriate outlets for grief expression [55].

There are currently six empirically supported interventions for bereaved youth which have demonstrated effectiveness in decreasing maladaptive coping, depressive symptoms, and posttraumatic stress symptoms [8]. Treatments include the Family Bereavement Program (FBP) [104], Grief and Trauma Intervention (GTI) [105], Trauma and Grief Component Therapy for Adolescents (TGCT-A) [106], Grief-Help [107], Trauma-Focused Cognitive-Behavioral Therapy (TF-CBT) [108], and Multidimensional Grief Therapy (MGT) [109]. While all interventions offer unique aspects, there are core components shared across treatments [110]. These include grief psychoeducation for children and caregivers, emotion identification and emotion regulation skills building, cognitive coping and restructuring, grief and trauma processing, memorializing and continuing bonds, meaning making, parental grief facilitation, future planning, and social support.

### 4.7. Trauma and Sudden Death

The circumstances of a child’s death (e.g., sudden versus anticipated death, peaceful versus violent circumstances) may have significant impacts on the grief and coping of their surviving family members. Siblings and parents alike may experience posttraumatic stress symptoms following a child’s death in addition to grieving their loss. Circumstance-related grief and trauma reactions impact children and adults differently; therefore, bereaved siblings and caregivers within the same family may have very different bereavement support needs. Adults bereaved by sudden (e.g., homicide, suicide, accident) rather than anticipated deaths (e.g., protracted illness) experience higher prevalence of prolonged or complicated grief [111,112,113].

For children bereaved under traumatic circumstances, intrusive symptoms (e.g., recurrent, unwanted images of the traumatic experience) and other post-traumatic stress reactions may interfere with positive reminiscing and adaptive grieving [114]. While traumatic deaths are often associated with violent and/or gruesome circumstances, research also demonstrates that repeated exposure to potentially traumatic events over time, such as seeing a chronically/terminally ill loved one experiencing a series of acute medical events and progressive physical decline, may also cause heightened post-traumatic stress.

Importantly, providers should not assume that because a death occurred under traumatic circumstances that surviving family members will be experiencing posttraumatic stress. Some research has demonstrated that complicated grief is predictive of distress and impairment after death beyond that which is accounted for by post-traumatic stress disorder (PTSD) [107,115]. Distinguishing between grief and posttraumatic stress symptoms is crucial in order to inform appropriate treatment. For example, it would be ineffective and potentially harmful to engage a bereaved sibling in completing a trauma narrative when their primary distress is related to missing their sibling and not PTSD [8].

### 4.8. Suicidal Thoughts and Behaviors

Bereaved caregivers commonly experience recurring thoughts of their own death, whether to be reunited with their child who has died or from the pain of living without their child. These thoughts can be quite normal for a bereaved parent [116]. The key to support is finding the balance between being alarmist and unconcerned [117]. Concern arises when these thoughts are coupled with intent, planning, or action. It is important to be aware of warning signs and equip bereaved people with the resources needed in times of suicidal crisis. In addition to support from family or friends, it is helpful for families to have contact information for a professional when the child dies to notify in the event of such thoughts. The professional might be the social worker, chaplain, doctor, grief and bereavement specialist, nurse, hospice, or other treating institution.

The professional can follow the National Institute of Mental Health recommendations for assisting persons who may be suicidal [118]: (1) ask if the person is planning on killing himself; (2) keep the person safe through reducing access to harmful objects; (3) listen to the person’s experience; (4) connect him with a local or national suicide hotline for triage; and (5) follow up with a supportive phone call after the crisis. A formalized suicide assessment through a hotline service can help the person get the help they need, particularly if the caregiver states a plan for suicide or has access to weapons. Alternatively, if the caregiver expresses passive suicidal ideation (“Sometimes I want to be with my child again” or “I don’t want to wake up some days”) but denies a plan, the professional can develop a safety plan and refer to a community mental health specialist along with the number to a local or national suicide prevention hotline for further care.

### 4.9. Disenfranchised Populations

There are groups of grieving people who are often disenfranchised by societal norms in their grief response [119]. The norms say: *This is not your death to grieve. You’re grieving too long. You’re grieving too much.* Consistently parents who experience miscarriage share experiences of unsupportive people minimizing their loss. When a child dies, the authors have seen many people pushed aside in their grief such as: step-parents, estranged family, adoptive or biological parents of children in protective services, LGBTQ parents, gestational carriers, extended family, classmates, teachers, and medical providers. It is the role of PPC to be inclusive and supportive to all individuals affected by the death. Programing should always enfranchise those grieving who have been told they should not. The death of a child effects many, and every person has the right to grieve that loss for as long as they need.

### 4.10. Longitudinal Programing

Grief has no time limit and there is no end to one’s grieving process; rather, people adapt to life without the child who has died. Therefore, PPC providers should offer longitudinal programing with services that encompass the first full year after a child dies. Families of children with chronic and life limiting illnesses often have a large support network of healthcare providers, friends, and community members while their child is alive, but such support regularly dissipates after funeral services [120]. As one parent stated, regarding longitudinal bereavement programing, “I am appreciative of the extended services. Everything cuts off after death except your services”. When possible, PPC providers should provide bereavement resources rather than outsourcing to facilitate a more personalized experience with healthcare team members who knew the child. When engaging in bereavement services, team members reference memories they built with the child and family. Various members of the PPC team (social workers, chaplains, Child Life Specialists, etc.) may offer support to different members of the family.

Sibling bereavement services are particularly important if the family was not willing to have discussions about prognosis and end of life with those siblings. These services are most impactful when offered face-to-face, allowing siblings to talk freely and engage in hands-on modalities. An initial session with a sibling or caregiver will determine the initial frequency of visits, which may be needed as often as weekly. As the sibling’s grief process progresses, the provider may taper bereavement services before finally discussing plans for “graduation”. A graduation session may include a personalized graduation certificate and activity of the sibling’s choice for the last session along with the bereavement care provider focusing on all the coping skills they have learned during their bereavement services.

Overall, longitudinal bereavement support is beneficial for both caregivers and siblings as it allows professionals to continue support as caregivers’ support from family, friends, and community may conclude [121]. One parent spoke to continuation of care, stating, “After a few months, people stopped asking about (child’s name) and saying her name and it became awkward if I brought (child’s name) up in conversation. It was always nice to be able to talk about her and understand that my experiences were normal and to have a place to talk about her without any fear.”

## 5. Innovative Approaches to Grief Support

### 5.1. Dignity Therapy in Pediatrics

**Figure 1:**
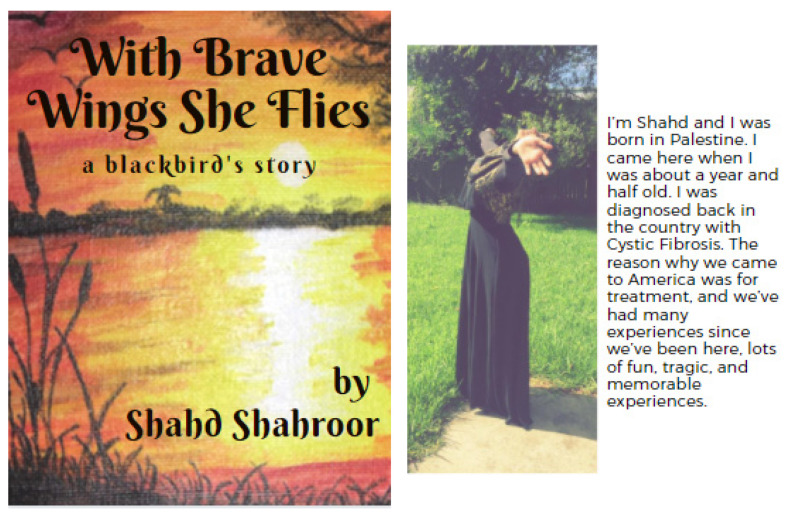
Printed with permission from Shahd Shahroor. Original art and photograph.

Schuelke and Rubenstein published a case series applying the adult version of dignity therapy to the pediatric population [122]. Created by Harvey Chochinov and colleagues as an intervention to support dying adults in their final days, the dignity therapy intervention reaffirms the person’s value and worth [123]. The process entails an interview with the dying person, editing of a transcript, creation of a story document, and recitation back to the dying person. The outcome is a paper-bound copy of the person’s life and wisdom to be gifted to an identified loved-one. Slight modifications to the interview and document editing make this intervention possible with adolescents, children, and proxies for preverbal or nonverbal children. Utilizing art and photographs from the dying child adds a layer of personalization to the final document (Figure 1). Application of dignity therapy with children is occurring all over the world, including the United Kingdom and Portugal [124,125]. A Family Dignity Intervention is being studied in Singapore [126]. Dignity therapy practices in PPC will most likely grow in the years to come.

### 5.2. Social Media Memorialization

The digital age brings new and innovative ways to incorporate memory of deceased children. Adolescents and young adults (AYA) with terminal prognoses are facing the conundrum of how to deactivate or set up their Social Media (SM) accounts as memorials [127]. Many teens find belonging and community through SM, where they cultivate close relationships. As one AYA asked, “What happens to my TikTok account? How will my Instagram followers know when I die?”

Addressing this important aspect of an AYA’s life can be validating and reassuring when providing advance care planning. Providers and AYAs may find creative ways to work within the AYA’s SM platforms for legacy building and death education. Some AYAs may choose to create stories and document their experience through SM, cultivating their personal image and legacy. The dying AYA may be self-conscious of their appearance or the way their voice sounds, so experimenting with voiceovers or music may be a sensitive way to address this concern.

### 5.3. Creativity in Legacy Work

Innovations in legacy work continue to evolve as PPC becomes more utilized with various populations. Working with families facing a life limiting in-utero diagnosis and legacy work can be very intangible in circumstances where multidisciplinary teams do not have physical access to the child until delivery. It is important to note that some of these women and their partners may have experienced the loss of at least one child/pregnancy previously, if not more, which perhaps can help the team ask about ways they might want to honor all their children. Perhaps the family was able to get ink prints from their other pregnancy losses and have memorial tattoos they would like to add, perhaps their goal is to get their infant home if possible, to spend time in their own environment. A newer legacy building item to consider for breastfeeding mothers is jewelry or keepsakes made from breast milk. Breast milk keepsakes can also include locks of hair, cremains and are able to be highly personalized.

Some families are not open to discussions about prognosis or death, which necessitates a more innovative approach to the types of legacy building referenced in Section 2.3. The following examples offer ideas for engaging such families in legacy work before the time of death. Perhaps these projects can be tied to certain holidays. For example, a child created a handprint canvas in the shape of a Christmas tree as this was their favorite holiday and surrounded the edges of the canvas with words that highlight their favorite things about that holiday during their time at the hospital. At the time of this child’s death their hospice team took note of an entire wall in the family’s home filled with this canvas and other artworks the child had created with their Certified Child Life Specialist during their time at the hospital. Additionally, heartbeat or voice recordings inside stuffed animals can be done in the context of giving them as a gift created by the dying child for their loved one in celebration of a holiday like Valentine’s Day or a loved one’s birthday.

Additionally, some children may have physical limitations that create difficulties for them to actively engage in traditional legacy building activities. Working with caregivers and siblings, providers can facilitate the ability for them to create tangible items such as having siblings or caregiver’s journal or facilitate writing stories about the dying child and what they loved, how they acted or stories about the care they provided for the dying child. Photos and videos can also be very beneficial for this population as these can help create scrapbooks and slideshows to honor the dying child [29]. For one sibling whose dying sister was bed-bound and had lengthy seizures that necessitated a minimally stimulating environment, the sibling’s favorite activity to engage with her dying sister was to read her books, particularly stories about princesses and to make silly faces and have her sister laugh and smile. This *intangible* legacy work was consistently repeated as a favorite memory by the sibling during longitudinal bereavement programing. Broadening the focus of keepsakes to include these intangible experiences of legacy propels PPC providers toward a more inclusive practice.

Innovative legacy building can also extend to the dying child’s participation in the planning of their funeral services [128]. For instance, maybe they want certain music played, a message from them read or they may create a slideshow of their favorite photos with their family and friends. One dying child wanted their family and friends to decorate their casket prior to their death and invited anyone attending their funeral to write them letters that their family could keep for their own memories. Another child wanted everyone to walk into their funeral to the Imperial Death March and had voiced wanting to literally “go out with a bang” and requested that there be fireworks at their burial which caused lots of laughter and fond memories to be shared at the graveside service.

Caregivers often look for new ways to find meaning and create legacy after their child has died. They might choose to ask for donations to advance research for their child’s diagnosis or plant a tree to grow and blossom over time. Some parents utilize creative writing to further their child’s legacy, like Antoinette Smith, mother of Marcus Smith who died in 2020. Readers can find Antoinette’s poetry in Appendix A, which was printed with permission. Others might change career paths or develop new nonprofit organizations in their child’s honor. An example of a creative legacy project following a child’s death can be found in Elise’s Legacy Library, an online tool for accessing mindfulness, deep breathing, guided imagery, and additional sessions to help children relax. A 20-year-old receiving cancer treatment at a pediatric hospital, Elise learned to manage her pain and anxiety with assistance from the music therapy and PPC teams through in-person music and guided imagery services. When she died, her mother worked with both teams to make Elise’s suggestion of providing these services to children outside the hospital a reality, saying “This program not only honors Elise for who she was, but also the teacher Elise wanted to be by teaching (patients) how to use these methods to feel better, too”. Creative programs like Elise’s Legacy Library serve to prolong the legacy of the child who died through improving the lives of others.

### 5.4. Telehealth

Research into telehealth has proliferated over recent years, highlighting the innovation and effectiveness of web-based psychological interventions. To date, there are seven published randomized controlled trials of web-based CBT interventions specifically designed for bereaved adults. Systematic review of these trials reveals moderate-to-large effect sizes in decreasing grief and posttraumatic stress symptoms with small effect size overall for reducing depressive symptoms, with treatment gains stable over time [129]. Of note, these trials include adults from various categories of bereavement (i.e., parents and non-parents, those bereaved by natural death versus violent death versus natural disaster, etc.).

Research has consistently demonstrated that effects of CBT-based intervention are comparable between in-person and web-based interventions [130]. Interventions designed for telemedicine range from self-guided and brief treatments to those with high levels of therapist involvement and feedback. Wagner and colleagues calculated an average attrition rate of 27% across trials, with highest dropout rates for treatments that were solely information-based (rather than interactive and reflective) and/or were briefer in treatment duration. While this attrition rate is comparable to other internet-based treatments, it is substantially higher than overall estimates of dropout from face-to-face interventions with children and adolescents (14.6%) as well as adults (17.5–19.2%) [118].

Despite a relatively higher attrition rate, web-based services allow families in need to access services that may otherwise be unavailable. In PPC, telehealth offers the opportunity for increased continuity of care from a child’s hospitalization at a tertiary care facility to inpatient or home-based hospice services. Avoidance behaviors are common in grief and asking families to return to the hospital where their lost child was treated may be an insurmountable ask. Web-based services allow these families to continue receiving care without needing to confront painful memories before they are ready. Depending on the state(s), telehealth services may also be permissible across state lines.

### 5.5. Virtual Memorials

The experience of planning and implementing memorial services has changed drastically since 2020. Many families are attempting to find innovative ways to bring their community together and provide a service for the child. One way memorial services have adapted to social norms is through the use of virtual memorials [131]. Family and friends can attend a service from their homes through the use of live streaming. Immediate family is present on-site with their child’s remains and many others join from afar. This has both comforting and distressing implications for immediate family. Nothing can replace physical touch and closeness, so to lose the tenderness of a caring embrace in a time of acute grief is very disconcerting. However, family that may not be able to attend otherwise (overseas, elderly, infirmed) are able to join the service. Virtual memorials can be a source of community and disconnection alike. Each family will need to examine what will be the most meaningful way to honor their child and have a community for grief.

### 5.6. International Collaboration

The ability to connect through social media platforms, online organizations, virtual conferences, etc. has created new and exciting potential for international collaboration, especially in regard to death education. Working together as a global-whole to advance the field of PPC is now more possible than ever before. Grief is often referred to as the universal language. However, cultural norms vary greatly. Collaboration beyond borders and oceans can help us all bridge the gap to better support our families in bereavement. The ability to connect is now endless, and the future is bright for comprehensive grief programing in PPC.

## 6. Conclusions

### 6.1. Summary

Current grief support practices with PPC families is multifaceted and personalized. Interventions and programing begins well before the death of the child, addressing non-death losses and anticipatory grief. The compassionate and intentional support around the time of death is crucial [132]. Bereavement support should be robust and tailored to each family’s needs. A variety of methods are helpful to families, i.e., cards, mail, calls, support groups, therapy, ceremonies, legacy building, and opportunities to make meaning. These supports continue to evolve as bereavement research provides recommendations and new grief theory emerges. Innovations in grief support will help foster a healthier coping environment for PPC families.

### 6.2. Barriers

The authors acknowledge that many organizations may not have the resources available to provide the comprehensive programing necessary to care for grieving families. There are many factors creating barriers within systems. First, the organization has to see the need and importance of supporting grieving families. Often these families are “no longer patients” after their child dies, and therefore the relationship is cut off. However, the literature and best practice are clear that the relationship can and should continue into the acute bereavement period, at the very least [133]. Institutions who care for their bereaved families often see a return of these families when their other children are ill. One parent said “The care we felt after my son died has made such a strong impression on me, I am only bringing my other children to this hospital in the future. I tell all of our friends and family to do the same”. Alternatively, families who feel unsupported in their grief have an opposite reaction, vowing to never return because they felt abandoned by the institution. This aspect alone supports creation of PPC grief programs, despite limited resources. Resources can always be found if the initiative is seen as important.

With limited resources, it is implied that there are not enough people to do this important work, despite the desire to provide appropriate programing. The authors acknowledge this moral dilemma and have empathy for this experience. Many institutions start with one person doing grief work for the entire system and/or multiple providers promoting grief support in addition to their assigned job descriptions. Praise and encouragement go to readers making a difference for grieving people in each way they are able. The authors would like to support your efforts in finding creative and innovative approaches to programing. It is incredible what a few dedicated people can create, causing lasting effects for grieving families. Inviting former bereaved parents to volunteer and support initiatives is a solid place to start [134]. Many past family members would love to support the newly bereaved and simply need a pathway through the medical system. PPC teams are a prime starting point for these types of initiatives.

Finally, a lack of education in death, dying, and bereavement (Thanatology) may create unforeseen barriers in programing. Organizations attempting to create services that are not based on up-to-date theory and practice run the risk of missing the mark for grieving families. Poor support can be worse than no support at all [135]. All PPC providers, regardless of discipline, should educate themselves in modern grief theory, supportive language, and best practices, or refer to experts for psychoeducation in grief. Utilizing grief education in daily practice is a helpful tool when working with families before, during, and after their child dies, and necessary for bereavement care programing.

### 6.3. Call to Action

If PPC teams hope for a more grief-healthy and death-educated world, promoting these services within daily practice is the beginning. Caring for each family’s grief throughout the trajectory of their child’s illness or injury sets the stage for promoting healthy coping in the future. Bereavement care is a meaningful and lasting component of the PPC spectrum of services and it cannot be overlooked. No longer should families feel abandoned by their medical teams. It is time to further our efforts in grief support.

## Figures and Tables

**Table 1:** Selected qualitative results from system-wide remembrance ceremony survey.

Question: Do you Have Anything You Would Like to Say about the Ceremony? Anything that Can be Improved?
“Beautifully done especially given the unprecedented circumstances of 2020. The program was beautiful.”
“Estaba trabajando, no pude verla, mis familiares que si la vieron estaban emocionados… Dios bendiga a todo el personal que hizo cuánto pudo por salvar ai hija, los llevo por siempre en mi corazón…” English Translation: “I was working, I couldn’t see it, my relatives who saw it were excited … God bless all the staff who did what he could to save my daughter, I carry them forever in my heart …”
“Every part of the program was meaningful and special to me. My heart and prayers go out to all the families who lost their beautiful child. I know that I’m not alone with the pain I feel everyday even though sometimes I feel I am. Thanks for remembering my sweet child who was only there a few hours.”
“Everything was absolutely beautiful. Thank you for all of your hard work.”
“I loved it. Thank you so much for doing this for my Angels and everyone else’s. This ceremony helps us to never forget.”
“I would like to see more personalized moments, not just the reading of names. All the generic stuff gets a bit old.”
“I would prefer lighting the candles at the beginning of the ceremony instead of closer to the end. The photo slideshow and descriptions were my favorite.”
“It was a great ceremony. Wasn’t sure what to expect but everything was very well done. Looking forward to experiencing in person.”
“Everything was absolutely beautiful. Thank you for doing such a wonderful job.”
“It was a sad but beautiful service. Thank you for your support and helping this hard process be a bit lighter to bare.”
“It was very meaningful and special. My heart and prayers go out to all those families who lost their beautiful child. I know the way I feel and thoughts I am not alone in forever pain. Thanks for remembering my sweet girl.”
“It was very moving to see some of our Palliative care team again. I would suggest adding additional medical team members be shown taking part. It warms my heart to see. At other hospital services, the care team taking care of the patient is asked to attend or participate. During a long illness, these care team members become your extended family.”
“Loved that you had each parent record their child’s name. I know that was very meaningful to us as grieving parents. Loved the program with pictures of each child—made me feel connected to the other parents and I enjoyed seeing pictures of all the other children.”
“Thank you all so much for putting this together. The ceremony was beautiful.”
“Thank you for making this event special. It was very organized and the package was very planned out. It was a nice event given the circumstances. Improvements….hopefully in person next year.”
“The moment the ceremony was over my husband looked over at me and said, ‘they always do such a great job’ with tears in his eyes. Feedback—I would prefer to light the candle at the beginning of the ceremony instead of at the end.”
“This program was very enlightening and help me and my family with our baby girl.”
“This was a beautiful ceremony and it was awesome to see all the young ones be honored and remembered.”
“You all did a great job ….we loved every minute of it. thank you all so much may God bless of your staff and as well as their family”

## Data Availability

No new data were created or analyzed in this study. Data sharing is not applicable to this article.

## Appendix A

Break Free. Do it for Me
by Antoinette Smith
20 January 2021
“Should my place at the table sit empty,
Should my voice in the halls of our home grow silent,
Should the warmth of my heart grow cold,
Should my sudden disappearance in the days of my youth
Seem to rob me of growing old,
Please know…
That my presence is still present in your memories.
I am still speaking through the life that I lived,
The people I touched, and the hope that that gives.
I’m still speaking, still reaching, still impacting—The ripples are still lasting.
My life may have been cut short in terms of time
But my legacy will live on well past the years of my prime.
You will always carry a part of me
And always remember the heart in me.
Let it drive you to keep moving, keep giving, keep growing.
Allow it to drive you to your destiny.
You cared so much for me
You must remember to care for you.
Keep in mind what I would have wanted you to do.
Give up? Stop living? No.
When a seed is sown
It has to be buried in order to grow.
Once the roots sprout out and push down
through the ground
A new stalk pushes up ignoring the weight of the dirt of the earth
Till it reaches past the surface.
Eventually in time
It should produce some type of fruit
For others to consume.
In that,
Life will keep on giving
So you too must keep on living.
I ran my race.
I finished my course
So why do you have remorse?
Pick up, repurpose, refocus on the reason you are still here.
Take a breath for me.
Conquer whatever your fears.
Break through the paralysis caused by your grief.
Break free.
Do it for me
And watch how you grow
Like that seed that was sown.
That’s what I would have wanted for you
And I desire for you to want that too.”
